# Effect of community – facility linked interventions on maternal health service utilization and newborn care in rural low-resource settings in Eastern Uganda

**DOI:** 10.1186/s12884-024-06883-4

**Published:** 2024-10-22

**Authors:** Solomon T. Wafula, Rornald Muhumuza Kananura, Gerald Pande, Felix Kizito, Sarah Namutamba, Betty Kyobe, Geraldine Agiraembabazi, Elizabeth Ekirapa-Kiracho, Peter Waiswa

**Affiliations:** 1 Department of Disease Control and Environmental Health, School of Public Health, College of Health Science, Makerere University, P.O Box 7072, Kampala, Uganda; 2 Department of Health Policy, Planning and Management, School of Public Health, College of Health Science, Makerere University, P.O Box 7072, Kampala, Uganda; 3 African Population and Health Research Center, Nairobi, Kenya; 4 Ministry of Health, P.O Box 7272, Kampala, Uganda; 5 Center of Excellence for Maternal, Makerere University, Newborn and Child Health, Kampala, Uganda; 6 University of North Carolina - Greensboro, 1400 Spring Garden Street, Greensboro, NC USA; 7 Department of Public Health Sciences, Karolinska Institutet, Stockholm, Sweden

**Keywords:** Maternal, Newborn, Community health workers, Healthcare utilization, Uganda

## Abstract

**Background:**

Improving maternal and newborn care (MNC) in hard-to-reach areas is essential for accelerating progress towards sustainable development goals (SDGs). We implemented the “Communities in which Mothers and Newborns Thrive (COMONETH) project” in rural settings of eastern Uganda between 2017 and 2020 to reduce barriers to accessing MNC services. We evaluated the effect of the COMONETH intervention on enhancing the utilization of MNC services and the adoption of appropriate care practices in Luuka district, Uganda.

**Methods:**

We used a pre- and post-comparison design to measure the effect of a demand-supply linked COMONETH intervention on MNC indicators. We trained Community Health Workers (CHW) to educate and refer expectant mothers to health facilities when needed. We also showed videos to pregnant women on identification of pregnancy danger signs, mentored and simulated health workers with PRONTO, and improved obstetric surgery at the referral facilities. We assessed antenatal care (ANC), facility delivery, postnatal care (PNC), and newborn care practices. We used optimal full propensity score matching, and weighted logistic regression and then estimated average treatment effect on the treated (ATT) of the intervention on MNC outcomes on the odds ratio scale.

**Results:**

A total of 583 women at baseline and 619 at endline participated in the study. The intervention was associated with increased odds of attending 4 ANC visits (OR = 1.26, 95% CI = 1.07–1.49), 8 ANC visits (OR = 2.27, 95% CI = 1.06–4.82) and utilization of PNC services (OR = 1.40, 95% CI = 1.20–1.63). We did not observe a significant association between intervention and early ANC attendance (OR = 0.88, 95% CI 0.80–1.00) and facility deliveries (OR = 0.99, 95% CI = 0.93–1.06). The intervention strategy was associated with improvements in practices: delayed bathing (OR = 1.22, 95% CI = 1.06–1.40), putting nothing on the cord (OR = 1.42, 95% CI = 1.27–1.59) and wrapping of babies immediately (OR = 1.08, 95% CI = 1.03–1.14).

**Conclusions:**

The findings demonstrated the potential of a demand-supply linked intervention to improve MNC outcomes in low-resource settings and should be promoted in similar settings. Interventions that strengthen the quality of care at health facilities and bridge demand-side gaps can improve MNC practices and reduce morbidity and mortality in rural settings.

**Supplementary Information:**

The online version contains supplementary material available at 10.1186/s12884-024-06883-4.

## Background

Maternal and newborn health is crucial for the well-being of women, newborns, families, and the overall community. However, in many rural areas, the time of pregnancy and childbirth is filled with anxiety due to the significant risks it poses to the health and survival of both the mother and the unborn child. Globally, neonatal mortality rates decreased from 5.0 million in 1990 to 2.3 million in 2022 but this still accounts for 47% of under-5 deaths [1]. Similarly, maternal deaths decreased by 44% from 385 per 100,000 live births in 1990 to 216 per 100,000 live births in 2015 [2]. However, the burden remains high, particularly in low and middle-income countries (LMICs) where the majority of maternal and newborn deaths occur, often going uncounted. In Uganda, maternal and neonatal mortality rates have remained unacceptably high for a decade, with estimates of 189 per 100,000 live births and 22 per 1000 live births respectively [3]. Rural communities experience even worse newborn mortality rates, such as 34 per 1000 live births in Eastern Uganda [4]. These deaths primarily occur due to complications that arise during pregnancy and childbirth, many of which could be prevented through the use of safe delivery care services [5].

Addressing these challenges requires the scaling up of effective interventions that tackle barriers to maternal and newborn health service accessibility, utilization and quality of care [5, 6]. Emphasis on such innovative strategies is a key component of the investment case for Reproductive, Maternal, Newborn, Child, Adolescent and Healthy Aging (RMNCAH) Sharpened Plan for Uganda [7]. As part of these plans, community health workers are key players who in many studies, have supported the mobilization and sensitization of communities, emphasizing the importance of antenatal care, facility delivery, and postnatal care (PNC), leading to increased health facility utilization [8, 9]. The demand creation, availability, and quality of maternal and newborn care (MNC) services also influence health facility utilization. Improving the quality of care involves interventions such as strengthening health workers’ skills, management and leadership training, and addressing stock-outs of essential commodities and staff shortages as previously demonstrated. Antenatal care is essential for preventing maternal deaths, and models with at least eight contacts are recommended to reduce prenatal mortality and improve women’s experiences [10]. The PNC however, is often neglected, with cost and transportation barriers hindering access. Bridging this gap requires outreach and home visits by community health workers to provide PNC at the community level. Knowledge and practices related to newborn care among women also need improvement. Harmful practices, such as applying harmful products to the newborn’s cord or immediately bathing the newborn after birth, contribute to newborn deaths [11–13]. Furthermore, there is limited knowledge of how to prevent or recognize newborn illnesses and how to promote kangaroo mother care (KMC) and its benefits [14]. Strengthening prenatal care can increase awareness of potential complications and danger signs during pregnancy, while investments in skilled birth attendants, access to emergency obstetric care, and transportation infrastructure are critical for reducing maternal deaths [15, 16].

Incorporating community-based strategies, such as training community health workers (CHWs), has demonstrated positive outcomes including enhanced awareness of maternal and newborn health, recognition of newborn danger signs, and improved utilization of antenatal care (ANC) and facility delivery services [8]. Implementation of facility-based strategies, such as the training of healthcare professionals, provision of emergency obstetric care, supply of necessary equipment, drugs, and supplies, as well as facility refurbishment, has demonstrated favorable outcomes in promoting facility delivery [5]. To alleviate the above challenges with regards to utilization of life saving MNC services, we implemented the Community in which Mothers and Newborns Thrive (COMONETH) project in collaboration with the district and partners. The COMONETH was a 3-year project (2017–2020) [17] that used a participatory action research approach to tackle both demand- and supply-side constraints. Details of this intervention are provided under methods. In this paper, we evaluated the effect of community-facility linkage intervention on the utilization of maternal and newborn services and care practices in Luuka district, a low-resource setting in Eastern Uganda. By doing so, we hope to contribute to the achievement of the Sustainable Development Goals (SDGs) and provide evidence for the effectiveness of such interventions on MNC in similar settings around the world.

## Materials and methods

### Study setting, design, and population

**Fig. 1:**
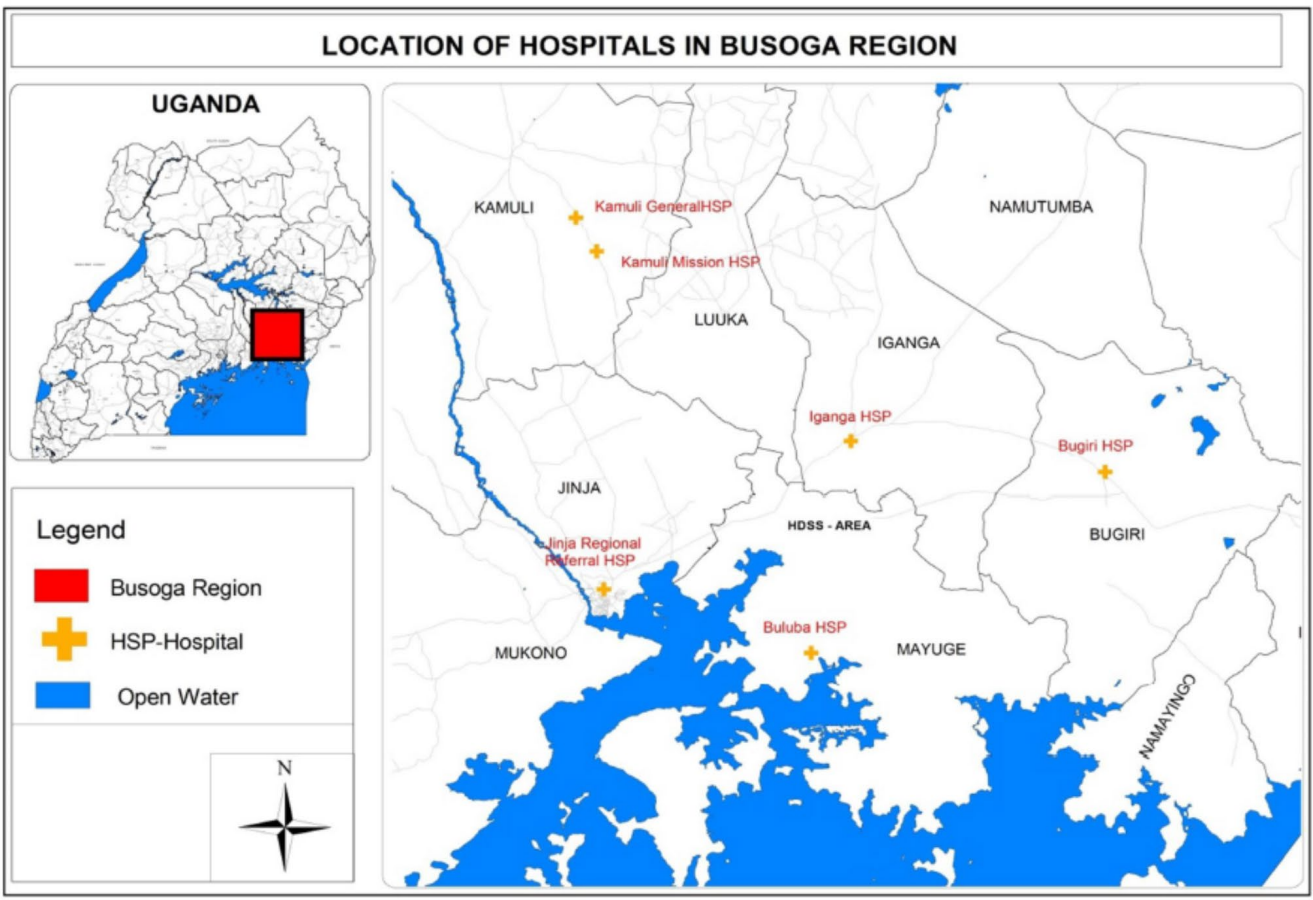
The map showing the location of Luuka and its neighboring districts and major hospitals in Busoga region

The study was implemented in Luuka district in in Eastern Uganda. Luuka is a hard-to-reach rural district and is bordered by Kaliro, Iganga, Mayuge, Jinja, and Kamuli districts. It has one county, eight sub-counties, and a population of 267,100 [18]. Luuka is a relatively new administrative district which was carved out of Iganga district in 2010. The district lacks a hospital but has 38 health facilities of which 28 are government owned and 10 are private facilities at the level of clinics (1), health centre IV (1), health centre IIIs (10) and health centre IIs (26) [19]. With limited health facilities, women in Luuka face challenges accessing services, living far from the nearest hospital in Iganga. The district’s population has grown steadily, and basic health and social services are primarily provided by the government and development partners [20]. The map of Busoga showing the Luuka, neighboring districts and major health facilities in the region is shown in Fig. 1.

This study employed a pre-test–post-test design study. A baseline cross-sectional survey was conducted among mothers with babies ≤ 12 months old, followed by an intervention and then another cross-sectional follow-up survey at the end of the intervention in the third year (2020). The study was conducted in all eight sub counties of Luuka district. Participants included women aged 15–49 years, who had recently given birth/ delivered and those with children aged one year and younger. These women (including the emancipated minors) had to be residents and able to consent. Those with severe illness or who refused to consent were excluded.

### Description of demand-supply linked COMONETH intervention

The COMONETH project, a community – health facility linked demand-supply intervention was implemented in Luuka district from September 2017 to September 2020 with funding from Comic Relief. It aimed to enhance the utilization of maternal and newborn health services as well as to promote quality care and safe practices during prenatal and neonatal periods. This would consequently mitigate the risk of maternal deaths, stillbirths and neonatal deaths. It was implemented across all sub counties of Luuka district. It had the following pillars: demand creation and referral, community engagement, supply enhancement, capacity building and sustainability as highlighted below:

**Creation of demand and referral** was achieved by working with the district to improve demand for maternal, newborn and child health care in the entire district. Various strategies were used which included skills enhancement of community health workers (CHWs) through quarterly support supervision. We enhanced their ability to carry out effective home visits, identify high-risk mothers and newborns, make referrals and fill out monthly reporting forms accurately. Special attention was paid to women with high-risk pregnancies; who were then supported to deliver at health centres by linking them to a voucher run by Marie Stopes on behalf of the Ministry of Health.

#### Community engagement

The project carried out community video shows in the local language (*Lusoga*) on appropriate maternal and newborn care practices in all villages in Luuka district, using existing “community video halls” (locally known as bibanda). The project also continued learning from every death by carrying out community dialogue linked to a verbal and social autopsy (VASA) process. Learnings from each death included understanding causes, and identifying care-seeking delay pathways. The CHW network engaged health workers to conduct integrated child health activities in the district, which enhanced district performance on MNC indicators.

#### The supply side

The district acquired an ambulance during project implementation, which increased health facility usage and enabled caesarean sections at the apex unit. Quarterly support supervision visits to the health facilities for progress review and hands-on mentorship for the health workers.

#### Sustainability and capacity building

To ensure sustainability, the project built a strong partnership model with health facilities, the district and communities encouraging their full participation. The CHWs with previous MNC experience were recruited and trained based on experiences from a previous study in Iganga district [9]. The CHWs supported demand creation as described above. The project also implemented on-the-job mentorship and PRONTO-based refresher training for healthcare workers to enhance care quality. PRONTO^SM^ (*Programa de Rescate Obstétrico y Neonatal: Tratamiento Óptimo y Oportuno*), is a simulation-based skills training for obstetric and neonatal emergencies [21, 22]. The trainings involved how to conduct safe deliveries, identification of danger signs, care for high-risk babies, and also strengthening managerial skills. In addition, the project hired a full-time medical officer and anaesthetic officer to make the theatre operational in providing emergency obstetric care services like caesarean section and newborn care services. After the project, the district and Ministry of Health (MOH) took over and maintained the recruited staff. The theatre runs well. MOH also provided an ambulance to the district which continues to serve the people.

### Variables and measurement

The primary outcomes examined in this study were initiation of antenatal care (ANC) within the first trimester, at least 4 and 8 ANC consultations, health facility delivery, postnatal care (PNC) within 6 weeks, and practicing recommended newborn care behaviors such as clean cord care /applying nothing, initiation of breastfeeding within one hour, cutting cord with a clean blade, and bathing newborn ≥ 24 h after birth (delayed bathing). The main exposure was the Intervention (represented by a period of time i.e., baseline (code 0) vs. endline (code 1)).

For this association between the intervention and the outcomes, various covariates were considered, including sociodemographic and individual characteristics such as age, educational level, parity, wealth and maternal knowledge of newborn care practices. Education level was recorded as “no education”, “primary”, or “post-primary”. Marital status was collapsed into “married/living with a partner”, and not “married”. Occupation was classified as “peasant farmer”, “housewife”, “business and other occupation” while parity was categorized into “primiparous”, “2–4”, or “5 and more”. Socioeconomic status (SES) (measured using asset index): Wealth quintiles were generated using principal components analysis (PCA) based on the assets and household structure data collected per the Uganda Bureau of Statistics (UBOS) criteria [20].

### Sample size and sampling procedures

For the household survey, the sample size was calculated in Open Epi based on the formula by Dean, Sullivan & Soe [23] as shown.

*N* = [DEFF*Np(1-p)]/ [(d2/Z21-α/2*(N-1) + p*(1-p)].

Where;

N = sample size that we require.

Np = Population size of the district.

P = estimated proportion of facility delivery was estimated (50%).

DEFF = Design effect (1.5).

Z = level of significance (95%).

This resulted in a total sample size of approximately 600 women. This means 600 participants at baseline and 600 at endline.

Regarding sampling at baseline and endline, a multi-stage sampling procedure was employed with the district as the survey domain. This assumed that all the sub counties within the district were homogenous in terms of health services utilization/accessibility, culture and geographic location. Multilevel sampling was done at the levels of parish, village and household. Sixteen parishes (2 parishes from each of the 8 sub-counties) were randomly selected; 2 or 3 villages were then randomly selected from each of the selected parishes and finally, between 12 and 20 households were randomly selected from each village (proportionate to size).

From the household listing exercise, a total of 4,740 households were found to have women who had babies below the age of 1 year. Of these, a sample of 583 were selected at baseline and 619 households at endline. It should be noted that we selected women who had babies below the age of 1 year since we needed information about recent pregnancies (pregnancy in the last 12 months) but also experiences with births and newborn care practices to counter recall bias. A breakdown of the numbers of women selected from each village is shown in supplementary file S1. Table 1.

### Data collection

The CHWs together with the local council representative supported data collection by providing administrative clearance and actively assisting researchers in navigating the community landscape. Data were collected in the local language from the mothers in selected households through face-to-face interviews using interviewer-administered questionnaires in 2018 and 2020. The tools were developed based on reviewed literature on maternal and newborn care services and community-based approaches [4, 6, 8, 9]. Data were collected on sociodemographic characteristics (age, parity, household size highest education level, wealth index, occupation, and marital status) and places of birth, number of ANC and PNC attendances, pregnancy gestational age at the first ANC visit, birth preparedness, and newborn care practices. The questionnaires were filled in by experienced Research Assistants (RAs), who were well-trained in objective interviewing to minimize recall bias from the mothers. After interviewing respondents, each RA compiled their completed questionnaires and handed them over to supervisors who reviewed the tools, checked for errors, and did the compilation.

### Data management and quality control

Before the baseline and endline surveys, research assistants underwent training. They followed a data collection manual that provided instructions for data collection, storage, and entry. To ensure accuracy, field supervisors conducted random re-interviews with selected respondents (about 20 re-interviews were conducted), while data editors checked for errors in the data collection forms. Any identified errors were promptly verified and corrected by the field staff. Additionally, an independent quality control team made weekly visits to the field to ensure adherence to the established protocol for data collection.

### Statistical analysis

Descriptive statistics were used to describe the baseline characteristics (age, gender, wealth index, parity, education level, occupation). Continuous variables were summarized as means and standard deviations and compared between baseline and endline using two-sample t-tests. Categorical data were expressed as frequencies and proportions and compared between baseline and endline using Pearson’s Chi-squared tests.

We conducted propensity score matching (PSM) to balance covariates between the intervention indicator groups (before and after) using the R package MatchIt [24]. We tested PSM using three different approaches: nearest neighbor, optimal matching and optimal full matching. The covariates included in the matching included age, education level, wealth index, parity, and receiving CHW visits. As there is no guarantee of balance after matching, we checked for balance in covariate distributions by treatment group before and after matching by visualizing the love plots. Our balancing criteria for the love plots were the standardized mean difference (SMD) values between − 0.1 and + 0.1 in line with Austin [25] and variance ratios (VR) balance between 0.8 and 1.25 [26]. Optimal full matching approach was chosen since it demonstrated superior covariate balance as compared to the nearest neighbor and optimal matching options.

Following matching, PSM weights were incorporated into the quasibinomial regression model object (without assuming interactions). The outputs of these regressions are highlighted in the supplementary file: S2. Tables 1, 2 and 3. We then estimated the average treatment effects on the treated from the model object, ATT (our target estimand) but also the average treatment effect in the population (ATE) assuming interactions between exposure with all covariates. The ATE was also reported because it is useful for cost-benefit analysis for population-wide policy. The resulting Odds ratios (OR) and 95% confidence interval have been reported. Analyses were performed in R version 4.3.1 software.

## Results

### Characteristics of participants

A total of 1202 mothers participated in this study (583 mothers at baseline and 619 mothers at endline). One-third 381(31.7%) were aged between 20 and 24 years (26.9% at baseline and 36.2% at endline). The median age of the respondents at baseline was 26 (IQR = 22,30) years (27 (22,30) years in baseline and 25 (21,30) years at endline). One-third, 390 (32.4%) of the mothers had attained post-primary education (Table 1).

**Table 1:** Sociodemographic characteristics of participants at baseline and endline surveys

Characteristic	Overall, *N* = 1,202^1^	Baseline, *n* = 583^1^	Endline, *n* = 619^1^	*p*-value^2^
**Age**				0.362
Median (IQR)	26 (22, 30)	27 (22, 30)	25 (21, 30)	
**Age in years**				< 0.001
15–19	126 (10.5%)	65 (11.1%)	61 (9.9%)	
20–24	381 (31.7%)	157 (26.9%)	224 (36.2%)	
25–29	332 (27.6%)	190 (32.6%)	142 (22.9%)	
30–34	194 (16.1%)	83 (14.2%)	111 (17.9%)	
35 +	169 (14.1%)	88 (15.1%)	81 (13.1%)	
**Education Level**				0.012
None	60 (5.0%)	36 (6.2%)	24 (3.9%)	
Primary	752 (62.6%)	379 (65.0%)	373 (60.3%)	
Post-primary	390 (32.4%)	168 (28.8%)	222 (35.9%)	
**Parity**				< 0.001
<=3	528 (47.8%)	202 (41.6%)	326 (52.7%)	
>=4	576 (52.2%)	283 (58.4%)	293 (47.3%)	
**Household SEP**				< 0.001
Poorest	230 (20.0%)	206 (35.6%)	24 (4.2%)	
Poorer	230 (20.0%)	152 (26.3%)	78 (13.7%)	
Moderate	230 (20.0%)	101 (17.4%)	129 (22.6%)	
Richer	230 (20.0%)	80 (13.8%)	150 (26.3%)	
Richest	229 (19.9%)	40 (6.9%)	189 (33.2%)	

^1^n (%)

^2^p-values from the Wilcoxon rank sum test or Pearson’s Chi-squared test as deemed appropriate,

For SEP, PCA scores were divided into quintiles

### Maternal and newborn health-related characteristics of respondents

In general, 572 (98.8%) and 614 (99.2%) of the participants had received at least one ANC visit at health facilities in the baseline and endline surveys, respectively. Overall, at least one ANC consultation remained consistently high throughout, with no significant differences observed. However, there was a significant increase in 4 + ANC visits from baseline (65.4%) to endline (78.0%), indicating improved utilization of antenatal care services. Health facility delivery also showed a slight increase from baseline (85.6%) to endline (89.8%). In terms of newborn care practices, there was notably a significant increase in participants putting nothing on the cord from baseline (68.4%) to endline (94.3%) (Table 2).

**Table 2:** Maternal and newborn health-related characteristics

Characteristic	Overall, *N* = 1,202^1^	Baseline, *n* = 583^1^	Endline, *n* = 619^1^	*p*-value^3^
ANC consultation [at least once]	1,186 (99.0%)	572 (98.8%)	614 (99.2%)	0.486
Early ANC consultation	921 (77.1%)	494 (85.0%)	427 (69.7%)	< 0.001
4 + ANC visits	859 (71.9%)	380 (65.4%)	479 (78.0%)	< 0.001
8 + ANC visits	57 (4.8%)	19 (3.3%)	38 (6.2%)	0.018
Health facility delivery	1,054 (87.8%)	499 (85.6%)	555 (89.8%)	0.026
Skilled birth attendance	1,064 (88.6%)	505 (86.6%)	559 (90.5%)	0.037
Mothers PNC	585 (48.8%)	157 (27.0%)	428 (69.5%)	< 0.001
Putting nothing on the cord	978 (81.8%)	397 (68.4%)	581 (94.3%)	< 0.001
Cord cut with a clean instrument	764 (63.7%)	337 (57.8%)	427 (69.3%)	< 0.001
Baby Wrapped in cloth immediately	1,015 (84.9%)	465 (80.3%)	550 (89.3%)	< 0.001
**Delaying bathing**				< 0.001
< 24 h	573 (48.0%)	310 (53.6%)	263 (42.8%)	
>= 24 h	620 (52.0%)	268 (46.4%)	352 (57.2%)	
Baby breastfed within 1 h	1,051 (87.7%)	496 (85.1%)	555 (90.1%)	0.008
Preterm birth	107 (8.9%)	60 (10.3%)	47 (7.6%)	0.102

^1^n (%)

^3^ p-values from Pearson’s Chi-squared test

### Estimated treatment (intervention) effects on the treated

The intervention was associated with increased odds of attending 4 ANC visits (OR = 1.26, 95% CI = 1.07–1.49), 8 ANC visits (OR = 2.27, 95% CI = 1.06–4.82) and utilization of PNC services (OR = 1.40, 95% CI = 1.20–1.63). We did not observe a significant association between intervention and early ANC attendance (OR = 0.88,95% CI = 0.80–1.00) and facility deliveries (OR = 0.99, 95% CI 0.93–1.06). The intervention strategy was associated with improvements in practices – delayed bathing (OR = 1.22, 95% CI = 1.06–1.40), putting nothing on the cord (OR = 1.42, 95% CI = 1.27–1.59) and wrapping of babies immediately (OR = 1.08, 95% CI = 1.03–1.14) (Table 3).

**Table 3:** Average treatment effects. The estimated marginal average treatment effects after fitting weighted regression are summarized in Table 3 below

	Average treatment effects in the treated, ATT (95% CI)	*p*-value	Average treatment effects in the population, ATE (95% CI) *	*p*-value
▪ Early ANC attendance	0.88 (0.79–1.00)	0.050	0.88 (0.80–1.00)	0.061
▪ Four ANC visits	1.26 (1.07–1.49)	**0.006**	1.25 (1.06–1.47)	**0.007**
▪ Eight ANC visits	2.27 (1.07–4.82)	**0.032**	2.12 (1.06–4.71)	**0.050**
▪ Health facility deliveries	0.99 (0.93–1.06)	0.813	0.99 (0.93–1.05)	0.713
▪ Postnatal care attendance	1.40 2(1.20–1.63)	**< 0.001**	1.39 (1.19–1.61)	**< 0.001**
▪ Putting nothing on the cord	1.42 (1.27–1.59)	**< 0.001**	1.41 (1.26–1.58)	**< 0.001**
▪ Delayed bathing	1.22 (1.06–1.40)	**< 0.005**	1.27 (1.10–1.46)	**< 0.001**
▪ Wrapping baby immediately	1.08 (1.03–1.14)	**0.003**	1.09 (1.03–1.16)	**0.003**
▪ Clean cord care	1.12 (0.98–1.28)	0.104	1.11 (0.97–1.28)	0.120
▪ Breastfeeding within an hour	1.04 (0.98–1.10)	0.235	1.05 (0.98–1.12)	0.156

Note*. For this analysis, we included interaction terms between intervention and all covariates

## Discussion

The findings indicate that a proactive demand-supply side district-wide intervention was effective in enhancing the utilization of maternal and newborn services and promoting positive practices throughout the stages of pregnancy, childbirth, and PNC. This approach resulted in increased attendance of four and eight ANC visits and postnatal care. Furthermore, notable improvements were observed in practices such as delayed bathing, adoption of clean cord care and PNC service.

### Effect of intervention on health facility utilization

Our findings indicate intervention was associated with improved ANC attendance (making at least four and eight ANC consultations). Home visits may have enhanced ANC clinic attendance by establishing trust, counseling patients, and encouraging appointment attendance, as well as assisting patients in overcoming any barriers to facility-level care. More ANC presents opportunities for early detection and management of complications, leading to a potential decrease in the severity of such complications and a subsequent reduction in the risks associated with maternal and infant mortality [8, 10]. Even while baseline ANC attendance (4 + visits) was slightly above the national average due to earlier, similar programs [8], there was a small improvement after the intervention. The noteworthy increase in coverage for these essential indicators is promising for such marginalized and remote communities. Similar interventions were shown to increase demand for ANC and led to improved attendance of at least four ANC visits compared to the baseline [8, 9, 27]. It’s however, important to acknowledge the potential effect of secular trends on the outcomes due to the lack of a comparison group hence cautious interpretation of findings is highlighted.

The promotion of institutional deliveries has been a key strategy for achieving maternal and newborn health goals. Our intervention, although an effect on facility-based deliveries was initially observed, the intervention did not demonstrate a significant effect after accounting for confounding. One reason for this could be that baseline facility deliveries were already high (86%) and only increased by 4%. It appears previous interventions may have had a lasting effect on facility deliveries [28]. These results suggest that once facility births increase, efforts should instead be focused on improving the content and quality of ANC, delivery and postnatal care. In addition, identification and support for high-risk pregnancies and a focus on who is left behind such as those with low education and those with fewer ANC visits becomes critical.

### Effect of intervention on newborn care practices and postnatal care

The intervention was associated with improved PNC attendance. PNC home visits are recommended to be conducted usually within a week of birth [29]. These visits play a significant role in promoting the implementation of essential lifesaving practices which can reduce neonatal mortality [5]. Our study revealed a notable increase in PNC visitations, something which has hitherto been difficult, suggesting that the intervention effectively enhanced both the capacity to demand and adhere to recommended PNC consultations.

Furthermore, maintaining hygiene during cord care is crucial for preventing infections in newborns [13]. We also found that the intervention improved cord care practices. Findings indicate that in some cases (especially baseline), cord cutting at birth was conducted using blades or scissors that were either used prior or were unclean. This poses a significant risk of infections, especially tetanus and HIV to both the mother and the newborn since there are high rates of tetanus [30] and HIV among pregnant women in this region [31]. The intervention resulted in an improvement in appropriate cord-cutting practices, increasing from 58 to 70% at the endline. However, a notable inverse association between institutional delivery and the use of new blades for cord cutting may suggest shortages of essential supplies such as blades. Ensuring quality care in facilities requires not only enhancing healthcare workers’ skills and providing leadership training but also ensuring adequate supplies [32]. As the number of facility births increase, efforts to ensure quality of care after deliveries through training, supervision, and the availability of essential medical commodities should be promoted by governments, districts and their partners.

Moreover, our findings demonstrated a notable 26% increase in the proportion of women who chose not to apply any substances to the baby’s cord (rising from 68 to 94%) following the implementation of the COMONETH intervention. It is worth noting that previous studies have shown that mothers frequently apply substances like ghee and ash to the cord, believing that it promotes faster healing and allows them to resume their household duties promptly [33]. Similar findings have been corroborated in other studies in Uganda [34], Tanzania [35] and Pakistan [36]. This initiative needs to be promoted to debunk such harmful practices which could endanger the health of newborns. Thus, engaging CHWs at the community level can be transformative.

Our study showed potential for intervention to improve delayed bathing and wrapping babies in warm clothing immediately after birth. WHO discourages immediate bathing of newborns after birth as it can result in health complications such as hypothermia and neonatal deaths [37]. Accordingly, bathing should be delayed until 24 h and where not possible for at least 6 h. Furthermore, it is also recommended to immediately wrap newborns with appropriate warm cloth and skin-to-skin contact to avoid hypothermia. Therefore, although the intervention reinforced the need to promote the implementation of evidence-based community-based interventions to enhance maternal and newborn health outcomes, gaps remain, and should be an intentional focus for CHW training and support supervision in future. If CHWs are well-trained to conduct antenatal home visits, this can contribute to better knowledge and management of newborn care practices, including delayed bathing and clean cord care [38, 39].

Although existing evidence highlights the importance of breastfeeding within 1 h [40], 15% of the mothers did not breastfeed their newborns within the first hour at baseline. This proportion marginally reduced to less than 10% following the intervention and ATE estimates were insignificant. Breastfeeding within 1 h has several benefits to babies including colostrum found in the first milk [41]. This practice was associated with higher knowledge of breastfeeding times for babies; an aspect which was also largely promoted as part of the overall intervention. We hypothesize that knowledge mediates part of the effect of the intervention on the practice of initiation of breastfeeding within an hour.

### Study limitations and strengths

This paper has a notable strength in its comprehensive approach to intervention, as it involves various stakeholders from health, transport, and politics. It also successfully brought together communities, local leaders, and experts to tackle the obstacles that impede the use of maternal and newborn health services. Among the limitations is that the intervention was a package consisting of several interventions and it was not possible to separate the effect of the specific interventions under the package. Potential recall bias since some data were obtained from mothers who had given birth almost a year before the surveys. Additionally, there is a chance of social desirability bias among the mothers, particularly when reporting their newborn care practices. Another concern relates to limited generalisability since the study was implemented in a predominantly rural district whose dynamics may be different from those in urban /affluent areas. Lastly, the before and after design without comparison group doesn’t allow full control of confounding and hence the findings should be interpreted with caution however we applied propensity score matching to minimize this bias. Despite its limitations, this study offers important insights into maternal health utilization and newborn care which is relevant for decision makers in Uganda and similar settings.

## Conclusion

The demand-supply side strengthening intervention improved utilization of maternal and newborn care in a poor rural district, which reinforces the need to scale up such proactive community-engaged health facility-linked interventions. As the number of facility births increase, efforts to ensure quality through training, supervision, and the availability of essential medical commodities should be ensured by governments, districts and their partners. In addition, CHWs can help to improve community maternal and newborn care practices. In addition, identifying and supporting those being left behind is critical. The combined efforts can result in reduced morbidity and mortality.

## Electronic supplementary material

Below is the link to the electronic supplementary material.

Supplementary Material 1

Supplementary Material 2

## Data Availability

The data is available from the corresponding author upon reasonable request.

## Abbreviations

ANC: Antenatal care
ATE: Average treatment effects
ATT: Average Treatment effect on the Treated
CHW: Community Health Workers
COMONETH: Communities in which Mothers and Newborns Thrive
KMC: Kangaroo mother care
LMIC: Low and middle-income countries
MNC: Maternal and newborn care
OR: Odds Ratio
PCA: Principal components analysis
PNC: Postnatal care
PRONTO: Programa de Rescate Obstétrico y Neonatal: Tratamiento Óptimo y Oportuno
PSM: Propensity score matching
SDG: Sustainable Development Goals
SEP: Socioeconomic Position
UBOS: Uganda Bureau of Statistics
WHO: World Health Organization
