# Supplementary material for: Effect of community – facility linked interventions on maternal health service utilization and newborn care in rural low-resource settings in Eastern Uganda
Source: BMC Pregnancy Childbirth. 2024 Oct 22;24:692. doi: 10.1186/s12884-024-06883-4 (PMC11515752; doi:10.1186/s12884-024-06883-4)
Supplement: Supplementary file 1 — Supplementary Material 1 [file 12884_2024_6883_MOESM1_ESM.docx]

**Supplementary files**

**S1. Table 1**: Household sampling summary per village at baseline and endline

| **Sub-county** | **Parish** | **Village** | **No. of households** | |
| --- | --- | --- | --- | --- |
|  |  |  | **Baseline** | **Endline** |
| Nawampiti | Buyoola | Buyoola | 17 | 18 |
|  |  | Ikonia C | 17 | 18 |
|  | Nakiswiga | Nakiswiga A | 19 | 18 |
|  |  | Buwamwa | 18 | 18 |
| Waibuga | Butimbwa | Ikonko | 19 | 19 |
|  |  | Lwanika | 19 | 17 |
|  | Itakaiboru | Buwiiri | 19 | 18 |
|  |  | Kigaya | 19 | 18 |
| Bukanga | Kiroba | Budoma | 12 | 12 |
|  |  | Bulonde | 12 | 12 |
|  |  | Nawandale | 12 | 12 |
|  | Budondo | Budondo A | 12 | 12 |
|  |  | Kimanto B | 14 | 12 |
|  |  | Budondo B | 13 | 13 |
| Ikumbya | Bunafu | Nawanyago | 16 | 18 |
|  |  | Bunafu A | 18 | 18 |
|  | Ntayigirwa | Idoome West | 19 | 18 |
|  |  | Nabitende | 18 | 18 |
| Bukooma | Bukyangwa | Budhana B | 14 | 18 |
|  |  | Bukyangwa B | 20 | 18 |
|  | Nabyoto | Nabyoto B | 19 | 18 |
|  |  | Buyoga | 17 | 18 |
| Bulongo | Bugonyoka | Nakisenyi A | 18 | 18 |
|  |  | Bugonyoka B | 19 | 18 |
|  | Namalemba | Busala | 17 | 18 |
|  |  | Bulike | 18 | 18 |
| Irongo | Irongo | Iganga A | 12 | 18 |
|  |  | Buwala | 12 | 19 |
|  |  | Naimuli B | 12 | 18 |
|  | Kibbinga | Nakavuma | 13 | 15 |
|  |  | Nkandakulyowa | 12 | 17 |
|  |  | Kibbinga A | 13 | 15 |
| Bulongo/ luuka TC | Lwanda | Bwambuzi | 18 | 16 |
|  |  | Lwanda | 19 | 16 |
|  | Kiyunga Ward | Kiyunga Industrial | 19 | 18 |
|  |  | Hospital Zone | 5 | 15 |
|  |  | Kiyunga Central | 13 | 20 |
| **TOTAL** | **16 Parishes** | **36 Villages** | **583 women** | **619 women/ households** |
