# Supplementary material for: Effect of community – facility linked interventions on maternal health service utilization and newborn care in rural low-resource settings in Eastern Uganda
Source: BMC Pregnancy Childbirth. 2024 Oct 22;24:692. doi: 10.1186/s12884-024-06883-4 (PMC11515752; doi:10.1186/s12884-024-06883-4)
Supplement: Supplementary file 2 — Supplementary Material 2 [file 12884_2024_6883_MOESM2_ESM.docx]

**S2 Table 1-3. Association between intervention and health service utilization indicators**

Before estimating ATE, weighted logistic regression results indicate that receiving the intervention was associated with 2.05 and 2.31 higher odds of attending at least 4 ANC visits (OR = 2.05, 95% 1.55 - 2.71) and 8 ANC visits (OR= 2.31, 95%CI 1.28 - 4.30) respectively. Furthermore, the findings show that participants who initiated ANC early had a significantly higher probability to complete 4+ ANC visits (0R =2.29, 95%CI 1.67 - 3.14) and 8+ ANC visits (OR =29.8, 95%CI 4.66 - 2,099) compared to those who did not initiate ANC early. We don’t observe a significant direct effect of intervention on early initiation of ANC and facility deliveries (**S2 Table 1)**

**S2 Table 1 Association between intervention and health service utilization**

|  | **Early ANC** | | **4+ ANC visits** | | **8+ ANC visits** | | **Facility delivery** | |
| --- | --- | --- | --- | --- | --- | --- | --- | --- |
| **Characteristic** | **OR**^1^ | **95% CI**^1^ | **OR**^1^ | **95% CI**^1^ | **OR**^1^ | **95% CI**^1^ | **OR**^1^ | **95% CI**^1^ |
| **Intervention (Time)** | 0.56 | 0.42 – 1.00 | 2.05 | 1.55 - 2.71 | 2.31 | 1.28 - 4.30 | 0.88 | 0.59 - 1.30 |
| **Age in years** |  |  |  |  |  |  |  |  |
| 15-19 | 1.00 | Ref. | 1.00 | Ref. | 1.00 | Ref. | 1.00 | Ref. |
| 20-24 | 0.84 | 0.44 - 1.52 | 1.73 | 1.00 - 2.96 | 0.41 | 0.14 - 1.36 | 3.40 | 1.70 - 6.75 |
| 25-29 | 1.08 | 0.54 - 2.09 | 1.23 | 0.69 - 2.21 | 1.47 | 0.50 - 4.88 | 1.81 | 0.88 - 3.68 |
| 30-34 | 0.41 | 0.19 - 0.85 | 1.10 | 0.56 - 2.15 | 0.27 | 0.06 - 1.21 | 2.76 | 1.15 - 6.69 |
| 35 + | 0.67 | 0.30 - 1.44 | 1.03 | 0.51 - 2.05 | 0.22 | 0.04 - 1.09 | 1.93 | 0.79 - 4.68 |
| **Parity** |  |  |  |  |  |  |  |  |
| <=3 | 1.00 | Ref. | 1.00 | Ref. | 1.00 | Ref. | 1.00 | Ref. |
| >=4 | 0.99 | 0.64 - 1.53 | 0.98 | 0.66 - 1.45 | 1.70 | 0.84 - 3.54 | 0.87 | 0.51 - 1.48 |
| **Education Level** |  |  |  |  |  |  |  |  |
| None | 1.00 | Ref. | 1.00 | Ref. |  |  | 1.00 | Ref. |
| Primary | 0.96 | 0.46 - 1.89 | 0.80 | 0.40 - 1.54 |  |  | 1.76 | 0.77 - 3.69 |
| Post primary | 1.09 | 0.50 - 2.24 | 1.04 | 0.50 - 2.10 |  |  | 2.23 | 0.91 - 5.14 |
| **Household SEP** |  |  |  |  |  |  |  |  |
| Poorest | 1.00 | Ref. | 1.00 | Ref. | 1.00 | Ref. | 1.00 | Ref. |
| Poorer | 1.43 | 0.89 - 2.33 | 1.23 | 0.81 - 1.88 | 1.89 | 0.69 - 5.59 | 0.40 | 0.22 - 0.71 |
| Moderate | 1.17 | 0.72 - 1.90 | 2.20 | 1.39 - 3.51 | 3.58 | 1.34 - 10.6 | 0.69 | 0.36 - 1.34 |
| Richer | 0.80 | 0.50 - 1.26 | 1.24 | 0.81 - 1.91 | 1.57 | 0.49 - 5.10 | 0.86 | 0.44 - 1.67 |
| Richest | 1.23 | 0.76 - 2.00 | 1.35 | 0.87 - 2.10 | 4.37 | 1.67 - 12.9 | 0.94 | 0.47 - 1.91 |
| **CHWs visits** | 1.01 | 0.74 - 1.37 | 1.11 | 0.83 - 1.48 | 0.57 | 0.31 - 1.03 | 1.75 | 1.18 - 2.61 |
| **Early ANC Initiation** |  |  | 2.29 | 1.67 - 3.14 | 29.8 | 4.66 - 2,099 |  |  |
| **EarlyANC** |  |  |  |  |  |  | 0.71 | 0.42 - 1.15 |
| **At least 4 ANC visits** |  |  |  |  |  |  | 1.53 | 1.01 - 2.30 |
| ^1^OR=Odds Ratios, CI = Confidence Interval,CHW= Community health worker | | | | | | | | |

***Note: We can see significant disparities in health facility deliveries by number of ANC visits and level of education (results not shown here). Targeting intervention for those with poor seeking behavior (<4 ANC visits) and low education is crucial***

**S2 Table 2. Association between intervention and newborn care services and postnatal care**

The intervention was associated with improved newborn cord care (putting nothing on the cord) (OR = 6.97, 95% CI = 4.75 - 10.40), delayed bathing (OR = 2.59, 95%CI 1.78 – 3.81) and use of PNC services (OR = 2.85, 95% CI = 2.05- 3.99). Moreover, knowledge about newborns being bathed after 24 hours exhibited a strong association with delayed bathing (**S2 Table 2**).

**S2 Table 2: Association between intervention and newborn care services and postnatal care**

|  | **Nothing on the cord** | | **Delayed bathing** | | **Postnatal care** | |
| --- | --- | --- | --- | --- | --- | --- |
| **Characteristic** | **OR**^1^ | **95% CI**^1^ | **OR**^1^ | **95% CI**^1^ | **OR**^1^ | **95% CI**^1^ |
| **Intervention (Time)** | 6.97 | 4.75 - 10.4 | 2.59 | 1.78 - 3.81 | 2.85 | 2.05 - 3.99 |
| **Age in years** |  |  |  |  |  |  |
| 15-19 | 1.00 | Ref. | 1.00 | Ref. | 1.00 | Ref. |
| 20-24 | 0.41 | 0.18 - 0.85 | 0.45 | 0.21 - 0.95 | 0.58 | 0.30 - 1.10 |
| 25-29 | 0.56 | 0.24 - 1.26 | 0.94 | 0.42 - 2.13 | 0.95 | 0.47 - 1.91 |
| 30-34 | 1.49 | 0.55 - 3.94 | 1.65 | 0.65 - 4.21 | 0.94 | 0.42 - 2.11 |
| 35 + | 1.52 | 0.56 - 4.06 | 1.66 | 0.63 - 4.37 | 0.81 | 0.35 - 1.87 |
| **Parity** |  |  |  |  |  |  |
| <=3 | 1.00 | Ref. | 1.00 | Ref. | 1.00 | Ref. |
| >=4 | 0.88 | 0.55 - 1.42 | 1.07 | 0.64 - 1.78 | 0.72 | 0.45 - 1.14 |
| **Household SEP** |  |  |  |  |  |  |
| Poorest | 1.00 | Ref. | 1.00 | Ref. | 1.00 | Ref. |
| Poorer | 1.68 | 0.95 - 3.02 | 1.15 | 0.64 - 2.07 | 1.30 | 0.78 - 2.18 |
| Moderate | 0.90 | 0.51 - 1.59 | 1.51 | 0.84 - 2.74 | 0.88 | 0.52 - 1.48 |
| Richer | 0.71 | 0.41 - 1.22 | 0.83 | 0.46 - 1.49 | 1.78 | 1.06 - 2.99 |
| Richest | 0.98 | 0.56 - 1.71 | 0.53 | 0.29 - 0.96 | 2.31 | 1.37 - 3.91 |
| **Education Level** |  |  |  |  |  |  |
| None | 1.00 | Ref. | 1.00 | Ref. | 1.00 | Ref. |
| Primary | 0.69 | 0.26 - 1.63 | 0.78 | 0.33 - 1.89 | 1.51 | 0.71 - 3.19 |
| Post primary | 1.53 | 0.55 - 3.90 | 0.82 | 0.33 - 2.07 | 1.45 | 0.65 - 3.21 |
| **CHW visits** | 1.29 | 0.90 - 1.86 | 0.79 | 0.54 - 1.17 | 16.6 | 11.7 - 23.9 |
| **At least 4 ANC visits** | 1.59 | 1.10 - 2.29 | 1.33 | 0.90 - 1.97 | 1.34 | 0.95 - 1.90 |
| **Health facility Delivery** | 0.77 | 0.42 - 1.37 | 0.91 | 0.53 - 1.57 | 0.63 | 0.38 - 1.03 |
| **Knows newborn are bathed after 24h** |  |  | 36.6 | 24.4 - 56.3 |  |  |
| ^1^OR=Odds Ratios, CI = Confidence Interval, CHW= Community health worker | | | | | | |

**S2 Table 3. Association between intervention and more newborn care practices**

The intervention was associated with improved newborn care practice of cutting the cord with a new blade” (OR=1.41, 95% CI=1.07-1.86), immediate “wrapping baby in cloth immediately after birth” (OR = 1.40, 95%CI 1.40 – 3.20) “initiating breastfeeding within the first hour of delivery (OR = 1.64, 95%CI 1.05 – 2.57) (**S2 Table 3**).

**S2 Table 3. Effect of intervention on newborn care practices**

|  | **Breastmilk within 1hour** | | **Immediate baby Wrapping** | | **Cut cord with new blade** | |
| --- | --- | --- | --- | --- | --- | --- |
| **Characteristic** | **OR**^1^ | **95% CI**^1^ | **OR**^1^ | **95% CI**^1^ | **OR**^1^ | **95% CI**^1^ |
| **Intervention (Time)** | 1.64 | 1.05 - 2.57 | 2.10 | 1.40 - 3.20 | 1.41 | 1.07 - 1.86 |
| **Age in years** |  |  |  |  |  |  |
| 15-19 | 1.00 | Ref. | 1.00 | Ref. | 1.00 | Ref. |
| 20-24 | 2.92 | 1.39 - 6.03 | 0.72 | 0.31 - 1.50 | 1.22 | 0.68 - 2.15 |
| 25-29 | 5.73 | 2.50 - 13.2 | 1.84 | 0.77 - 4.15 | 0.74 | 0.41 - 1.30 |
| 30-34 | 3.54 | 1.48 - 8.65 | 1.74 | 0.69 - 4.32 | 0.81 | 0.44 - 1.48 |
| 35 + | 0.94 | 0.43 - 2.01 | 0.65 | 0.27 - 1.48 | 0.30 | 0.16 - 0.55 |
| **Education Level** |  |  |  |  |  |  |
| None | 1.00 | Ref. | 1.00 | Ref. | 1.00 | Ref. |
| Primary | 0.51 | 0.14 - 1.50 | 0.48 | 0.14 - 1.33 | 1.08 | 0.55 - 2.08 |
| Post primary | 0.45 | 0.12 - 1.41 | 0.72 | 0.19 - 2.14 | 0.76 | 0.37 - 1.52 |
| **Knows breastfeeding times** | 13.4 | 8.05 - 22.7 |  |  |  |  |
| **CHW visits** | 0.82 | 0.51 - 1.30 | 0.80 | 0.52 - 1.23 | 1.36 | 1.02 - 1.82 |
| **Household SEP** |  |  |  |  |  |  |
| Poorest | 1.00 | Ref. | 1.00 | Ref. | 1.00 | Ref. |
| Poorer | 0.81 | 0.37 - 1.83 | 0.85 | 0.43 - 1.64 | 1.30 | 0.85 - 2.01 |
| Moderate | 0.41 | 0.20 - 0.83 | 1.55 | 0.73 - 3.40 | 1.31 | 0.84 - 2.04 |
| Richer | 0.44 | 0.22 - 0.87 | 0.58 | 0.30 - 1.08 | 1.83 | 1.18 - 2.85 |
| Richest | 0.62 | 0.29 - 1.31 | 0.81 | 0.41 - 1.55 | 1.61 | 1.04 - 2.50 |
| **At least 4 ANC visits** | 1.28 | 0.79 - 2.04 | 1.57 | 1.03 - 2.37 | 1.14 | 0.85 - 1.54 |
| **Health facility delivery** | 0.76 | 0.32 - 1.61 | 1.37 | 0.73 - 2.45 | 0.11 | 0.05 - 0.20 |
| **Preterm birth** |  |  | 0.49 | 0.26 - 0.98 |  |  |
| ^1^OR=Odds Ratios, CI = Confidence Interval, CHW= Community health worker | | | | | | |
